# Review of Recent Progress on Vertical GaN-Based PN Diodes

**DOI:** 10.1186/s11671-021-03554-7

**Published:** 2021-06-07

**Authors:** Taofei Pu, Usman Younis, Hsien-Chin Chiu, Ke Xu, Hao-Chung Kuo, Xinke Liu

**Affiliations:** 1 College of Materials Science and Engineering, Shenzhen University–Hanshan Normal University Postdoctoral Workstation, Shenzhen University, Shenzhen, 518060 China; 2 Key Laboratory of Optoelectronic Devices and Systems of Ministry of Education and Guangdong Province, College of Physics and Optoelectronic Engineering, Shenzhen University, Shenzhen, 518060 China; 3 Department of Electronic Engineering, Chang Gung University, Taoyuan, 333 Taiwan; 4 Suzhou Institute of Nano-Tech and Nano-Bionics, Chinese Academy of Sciences, Suzhou, 215123 China; 5 Photonic and Institute of Electro-Optical Engineering, National Chiao Tung University, Hsinchu, 300 Taiwan

**Keywords:** Gallium nitride, Vertical PN junction diode, Electrical field crowding, Edge termination techniques

## Abstract

As a representative wide bandgap semiconductor material, gallium nitride (GaN) has attracted increasing attention because of its superior material properties (e.g., high electron mobility, high electron saturation velocity, and critical electric field). Vertical GaN devices have been investigated, are regarded as one of the most promising candidates for power electronics application, and are characterized by the capacity for high voltage, high current, and high breakdown voltage. Among those devices, vertical GaN-based PN junction diode (PND) has been considerably investigated and shows great performance progress on the basis of high epitaxy quality and device structure design. However, its device epitaxy quality requires further improvement. In terms of device electric performance, the electrical field crowding effect at the device edge is an urgent issue, which results in premature breakdown and limits the releasing superiorities of the GaN material, but is currently alleviated by edge termination. This review emphasizes the advances in material epitaxial growth and edge terminal techniques, followed by the exploration of the current GaN developments and potential advantages over silicon carbon (SiC) for materials and devices, the differences between GaN Schottky barrier diodes (SBDs) and PNDs as regards mechanisms and features, and the advantages of vertical devices over their lateral counterparts. Then, the review provides an outlook and reveals the design trend of vertical GaN PND utilized for a power system, including with an inchoate vertical GaN PND.

## Introduction

Global energy consumption is rising much more rapidly than in the past few decades because of the rapid growth in industry and economy. Therefore, advanced energy-saving technologies are required to alleviate the issues of increasing energy consumption.

Silicon-based devices are currently the dominant type among power devices [1]. Among these power devices, the insulated gate bipolar transistor (IGBTs) structures play a significant role and display a growing trend toward the replacement of the power bipolar junction transistor (BJT) and metal oxide semiconductor field effect transistor (MOSFET) since the invention of the IGBTs in 1982 [2]. Presently, IGBTs are essential elements of power electronic fields for conversion and transmission [3]. However, silicon-based power devices have reached their fundamental material limits to date and are utilized widely in electric energy application.

Given its outstanding properties, GaN is one of wide bandgap semiconductor materials (including SiC, GaN, Ga_2_O_3_ and diamond) capable of fabricating power devices with a low capacitance and resistance for a specified breakdown voltage with respect to the Si-based devices. Thus, GaN-based devices with low energy consumption, high power densities, and high conversion efficiency for power electronic systems are expected.

As shown in Table 1, GaN has a much higher Baliga’s figure of merit (BFOM) than SiC (which has been commercialized), a feature which is ascribed to its higher electron saturation velocity and higher critical electrical field. However, SiC has better thermal conductivity. In addition, GaN possesses a higher carrier mobility *µ* and a higher thermal conductivity, and achieved both *p*- and *n*-type doping compared to Ga_2_O_3_. Meanwhile, for the ultimate wide bandgap semiconductor, diamond, considerable progress must be achieved to reach the practical stage. To date, GaN-based devices, e.g., three-terminal device (Heterostructure field-effect transistors (HFETs) and MOSFETs) and two-terminal devices (SBDs and PNDs) have become key research topics, and great progress has been achieved in their applications in power rectification and power conversion.

**Table 1:** Material parameters of Si, GaAs, 4H-SiC, GaN, Ga_2_O_3_, and diamond

Materials	*E*_g_	*ε*	*µ*_n_	*E*_c_	*V*_sat_	Thermal conductivity	BFOM
Si	1.12	11.7	1450	0.3	1	130	1
GaAs	1.42	12.9	8500	0.4	2	54	15
4H-SiC	3.26	10	950	3.0	2	500	340
GaN	3.39	9	1000	3.3	2.5	130	870
β-Ga_2_O_3_	4.8–4.9	10	300	8	2	20	3444
Diamond	5.6	5.7	4000	10	3	2000	24,664

*E*_g_ (eV), *ε*, *µ*_n_ (cm^2^/V s), *E*_C_ (MV/cm), *V*_sat_ (10^7^ cm/s) and BFOM (*εµE*_b_^3^, to Si) are energy bandgap, relative dielectric constant, electron mobility, critical electric field, saturation velocity and Baliga’s figure of merit, respectively, and thermal conductivity of (W/m K) [4, 5]

### GaN versus SiC

**Fig. 1:**
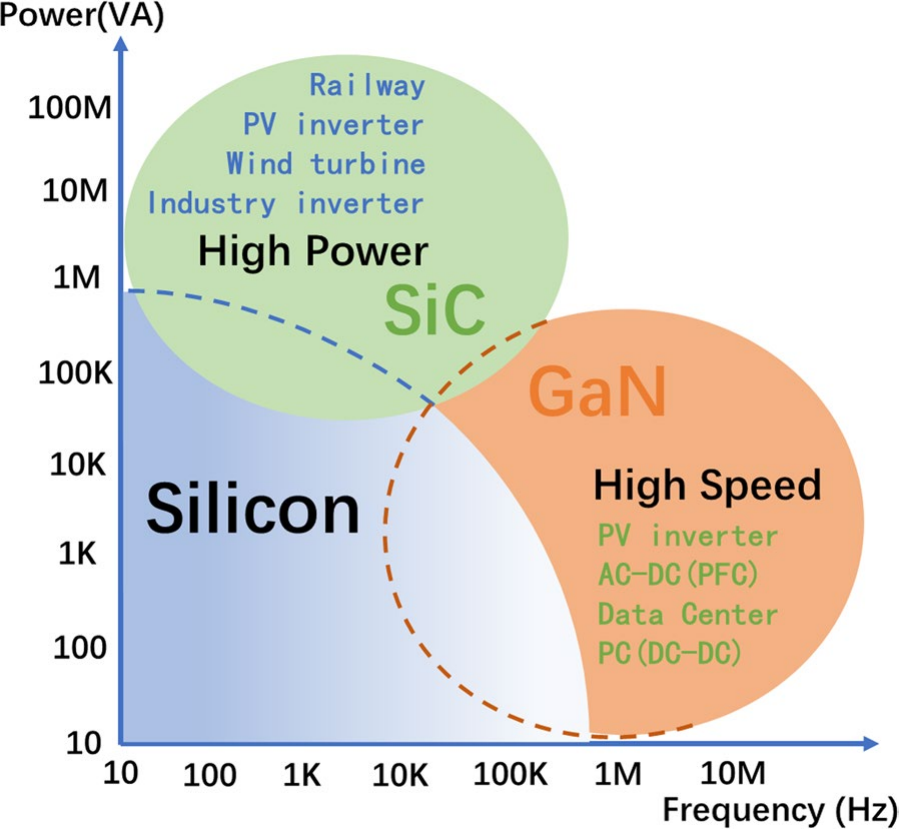
Potential applications of GaN and SiC power switching transistors [7]

From its inherent material properties, GaN has slightly superior advantages over SiC, including a wider energy bandgap, higher critical electrical field, higher electron saturation velocity, and a 3–4× better BFOM for power devices [6]. Thus, considering its outstanding features, GaN-based devices should outperform SiC-based counterparts. In practical application, vertical GaN-based devices are essential for high power density and high operating frequency (Fig. 1) because of their excellent material properties.

As the most mature wide gap semiconductors, SiC devices have achieved remarkable advances in the last decade and show promising performance in terms of high voltage, low specific on-resistance, and fast switching speed [8]. Given the profound research basis since 1980 and available larger SiC substrate with low defect (< 10^4^ cm^−2^), SiC SBDs and junction field-effect transistors (JFETs) were the first commercialized SiC-based devices since 2001. Other SiC-based power devices including MOSFETs and BJTs were successfully developed in the field of high voltage and power application and have demonstrated impressive performance [9].

In comparison with SiC, GaN devices developed very slowly. Their unsatisfactory material quality prevents the realization of some of their theoretically superior properties. Given the lack of the GaN substrate, most studies on GaN devices are mainly based on the lateral structure (e.g., AlGaN/GaN heterostructure) at this stage. With its higher electron mobility of 2000 cm^2^/V s (two-dimensional electron gas (2DEG) in AlGaN/GaN), 1000 cm^2^/V s (bulk GaN) and higher saturation velocity of 2.5 × 10^7^ cm/s relative to that of SiC counterparts, AlGaN/GaN devices are preferred for high-frequency applications and have low power watts with respect to SiC devices [10]. At high frequency fields, AlGaN/GaN SBDs attain excellent electrical transport performances and are more suitable for microwave and millimeter wave applications at the watt level [11, 12]. The AlGaN/GaN SBD is a representative device for GaN SBDs. The high mobility of 2DEG means that AlGaN/GaN SBDs demonstrate huge performance advantages at high-frequency applications relative to vertical SBDs and maintain a low turn-on voltage as well. Recently, AlGaN/GaN SBDs were the basis for a 5.8-GHz rectifier circuit with an input power of 6.4 W and a turn-on voltage 0.38 V with a breakdown voltage (*BV*) of 3000 V [13, 14]. The maximum cutoff frequency is close to 1 THz at 0 V with an anode-to-cathode distance of 70 nm [15].

For GaN-based power devices, the GaN substrate (bulk GaN) is ideal for epitaxy growth, which could utilize homo-epitaxy technology to eliminate the mismatch. The low dislocation density of bulk GaN is essential for the epitaxial substrate because the high dislocation density can affect performance characteristics such as *BV*, reverse leakage current, production volume, and reliability [16]. The developments of vertical GaN-based devices have been driven by the progress of the GaN substrate in recent years. However, given the relatively immature technology for the vertical triode, the vertical GaN diode has become a hot research topic at this initial stage. Compared to AlGaN/GaN SBDs, vertical GaN SBDs have similar advantages at frequency fields such as high switching speed with low reverse recovery time and low conduction loss; nevertheless, the latter has large current density and less leakage path than the former [17, 18].

Some issues have arisen for the GaN substrate. First, GaN substrates are currently available with dislocation densities of 10^4^–10^6^ cm^−2^, but these dislocation densities are still much higher than those of Si and SiC substrates [19]. Second, with respect to the 4–6 inch wafer size and reasonable cost (10 euro/cm^2^) of SiC, the 2–3 inch size and relatively high cost (100 euro/cm^2^) of the GaN substrate inhibit GaN commercialization and productivity on a large scale [20]. Aside from increasing the supplying vendors, the heteroepitaxy on foreign substrates (Si, sapphire, or SiC) is an alternative way to reduce the cost of GaN substrate, but the challenge of relatively high mismatch and defects must be addressed.

In addition to the aforementioned issues in bulk materials, the challenge for the GaN diode is to achieve a high quality of the *p*-type material. With respect to the relatively advanced techniques for forming *p*-type SiC by Al ion implantation, immature *p*-type ion implantation technology and the low acceptor activation for *p*-type GaN are vital hindrances, which limit the development of the structure and fabrication of GaN-based devices. Thus, different kinds of edge termination structures are proposed to improve the GaN diode performance and constitute a key part of this review.

### Vertical GaN-Based Devices on the FS-Substrate

Until 10 years ago, most GaN diodes, including SBDs and PNDs, were fabricated on foreign substrates forming lateral or quasi-vertical device structures because of the unavailability of a bulk GaN substrate. Lateral or quasi-vertical diodes show outstanding electrical characteristics, but they still suffer from inherent drawbacks from the device structure [21]. First, the mismatch and defects are inevitable. Then, for GaN devices on foreign substrates, a buffer is essential between the GaN drift layer and foreign substrate. Thus, the stress relaxation of the buffer layer would cause a wafer bowing, which will lower the thickness of the GaN epitaxy layers [22]. In addition, the large thermal boundary resistance (GaN with substrate) has a serious influence on device performance for the GaN devices working at high power density [23].

According to the development of epitaxy techniques, free-standing GaN (FS-GaN) substrate with low dislocation density adopted for vertical GaN devices has made great progress. The material quality of homoepitaxial GaN on the FS-GaN substrate has an obvious improvement and shows the potential for power applications. Vertical GaN devices could mitigate the drawbacks of lateral GaN counterparts. First, thicker GaN epitaxial layers can be grown without any buffer, and higher *BV* (which exceed that of lateral GaN devices) can be obtained and determined by the thickness of the drift layer. Meanwhile, a maximum electric field is located at the inner part of devices and is far from the device surface (eliminating the effect of electron trapping which generally occurs in lateral devices). Given the high quality of the FS-GaN substrate, vertical GaN devices have been studied since 2011 [24]. Since then, vertical GaN PNDs with high *BV* and low on-state resistance have been fabricated and achieve remarkable performance.

### PND versus SBD

**Fig. 2:**
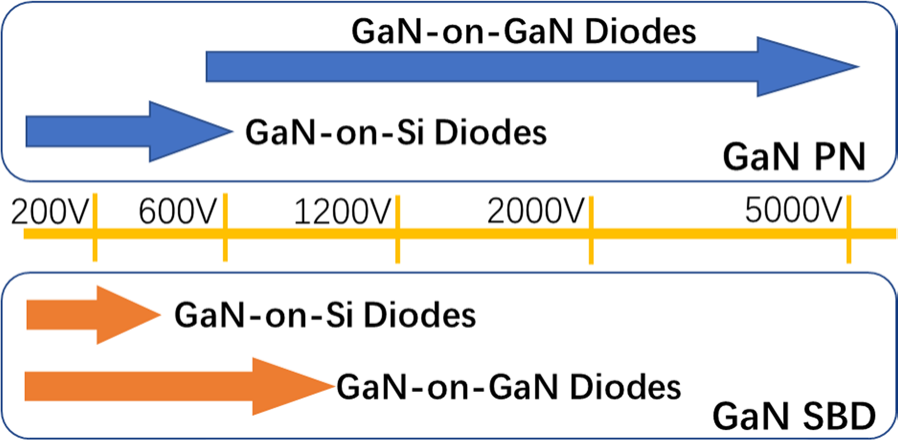
Overview of the device types, reports, and voltage classes of main vertical GaN power devices reported in recent years [26]

For practical applications, diodes are essential components for power conversion and inversion [25]. With the distinct material properties of GaN, GaN-based diodes (SBDs and PNDs) exhibit notable performances, which are expected to satisfy the requirements of power applications. As shown in Fig. 2, the PNDs have the largest scope among GaN devices with varied structures and withstand voltages from 600 to 5000 V, a feature that indicates wider application scenarios.

Compared with PNDs, which have no minority carrier storage issue and lower SBD barrier height, GaN SBDs feature a low forward turn-on voltage (*V*_on_) and fast reverse recovery, and these characteristics indicate that the merits of SBD are revealed in low conduction/switching loss, high-frequency operation, but a lower *BV* value than that of PND; moreover, note that high turn-on voltage can lead a high conduction loss and degrade the efficiency of circuits and systems [27, 28].

At the high-power fields, quasi-vertical or vertical SBDs have excellent advantages over AlGaN/GaN SBDs [29]. Moreover, a low turn-on voltage (< 0.5 V) is achieved by adjusting the barrier height with a low work function metal. However, a low barrier height may easily lead to the high reverse leakage current and lower the *BV*. Consequently, varied edge termination structures are proposed to improve performance. Through the assistance of varied edge termination technologies [27, 28, 30, 31], the high current density at KA/cm^2^ grade with *BV*s over 1 kV is confirmed. Meanwhile, vertical SBDs also exhibit great capability for high frequency, such as 177–183 GHz and a maximum of 902 GHz cutoff frequency at 0 V, a feature which is expected for power sources in terahertz-wireless communication systems [12, 32].

With respect to *n*-type GaN, the *p*-type GaN grown by metalorganic chemical vapor deposition (MOCVD) and molecular beam epitaxy (MBE) was introduced by using Mg as an acceptor [33]. Compared with GaN SBDs, GaN PNDs have many advantages, such as low specific on-resistance (*R*_on_*A*) and adequate device stability and capability of inhibiting surge currents. Despite the high turn-on voltage (> 3 V) and relatively low switching speed, an ultralow leakage current because of the higher barrier height and high *BV* is obtained. Note that a high *BV* from 0.6 to 5 kV is the most outstanding performance for GaN-based PNDs. Thus, GaN-based PNDs have great potential as important building blocks of the next-generation power systems for high power applications, which require high efficiency and low energy loss [34]. Similar to vertical GaN SBDs, the edge terminations for GaN PNDs are also fabricated to alleviate electric field crowding around anode, a feature that would be described in detail later in this article.

In power devices, the PN junction can be also treated as a novel junction structure. For utilizing a high *BV* with low reverse leakage current of PNDs, high switching performance and low turn-on voltage of SBDs at the same time, novel device structures including the junction barrier Schottky (JBS) and merged PN Schottky (MPS) generate a combination of SBD and PND by the formation of *p*-type grid regions in the Schottky contact region. JBS or MPS devices possess the characteristics of forward conduction of SBDs and reverse blocking of PNDs and are expected to have better switching performance and higher reverse voltage than conventional PNDs and SBDs, respectively [35].

Besides their high-power application, GaN PNDs are also adopted for optoelectronic devices such as light-emitting diodes (LEDs), photodetectors, and for flame sensing because of the low dark currents of a few pA in GaN-based PN junction photodiodes [36, 37].

### Purpose of This Review

On the basis of the superior material properties of GaN, GaN-based devices have been investigated widely and utilized in power electronics applications as the one of the hottest semiconductor materials. Among GaN-based devices, the vertical GaN-based PND has been considerably explored and shows excellent BFOM. The edge terminal techniques are also essential to alleviate the electric field crowding around the anode pad.

In this review, the advances in material epitaxy growth and edge terminal techniques as the main emphases are followed by illustrating the current GaN developments, the differences between GaN SBDs and PNDs in terms of mechanisms and features, and the advantages of vertical devices over lateral one. This review provides an outlook and reveals the design trend of vertical GaN PNDs utilized for a power system, including inchoate vertical GaN PNDs. The development of epitaxial growth corresponding to a different layer in vertical GaN PNDs is introduced in Sect. 2. The edge terminal technologies are explored in Sect. 3. In Sect. 4, the vertical GaN PNDs on Si substrate are demonstrated as an alternative method. Finally, the conclusion and outlook of future development of vertical GaN PNDs are provided.

## Material Epitaxy Growth

### GaN Substrate of Vertical PNDs

As a mainstream epitaxial process, vertical device epitaxial layers are currently mainly grown by MOCVD on conductive GaN substrates fabricated by hydride vapor phase epitaxy (HVPE). In this structure, the quality of the substrate directly affects the following epitaxial structure. Many failure mechanisms in the device originate from substrate epitaxy quality [38]. A high quality of conductive GaN substrate must be obtained to further improve the forward and reverse *I*–*V* performances, especially the reverse leakage current and *BV* capability.

Substrate grown by HVPE has been considered as a most convenient method for mass production because of its relatively low cost and reproducibility. However, in the early stage, the immature growth technology meant that GaN substrate grown by HVPE had high background carrier concentration (> 10^19^ cm^−3^) and unsatisfactory crystal quality. Therefore, the HVPE was not adopted to grow the device structure [39]. By the rapid development of epitaxy growth technology, high-quality bulk GaN materials by HVPE become gradually possible [40, 41]. Aside from the substrate, the partially epitaxial layer in device can now be grown by HVPE and can result in higher current uniformity and the elimination of the macrostep on the GaN surface by combining HVPE and the MOCVD epitaxial process with carbon-free technology compared devices grown solely by MOCVD [42].

Currently, a commercial GaN substrate with minimum dislocation density of less than 10^4^ cm^−2^ is available. The GaN vertical PNDs on low defect density GaN substrate were processed. However, wafer bowing remains an issue. As a promising solution, the ammonothermal method can achieve a GaN substrate with better epitaxy quality. With the high-pressure autoclaves and supercritical ammonia, the threading dislocation can be significantly reduced to 10^4^–10^5^ cm^−2^ [43]. Using the ammonothermal method, a low resistance of 0.001 Ω cm^2^ in the GaN substrate with highly doped *n*-type (1 × 10^19^–1 × 10^20^ cm^−3^) was reported as well as the *BV* of 3 kV [44]. However, the drawback of the ammonothermal method is the low growth rate of approximately 80–90 μm/day, a feature that is adverse for practical production. Improving the growth rate and maintaining high material quality are also profitable directions for subsequent research.

In addition to the commercial GaN substrate, some substrates were also investigated for distinctive performances, including the non-Ga-plane (*c*-plane) substrates. Generally, a Ga-polar (i.e., *c*-plane) substrate is employed for a GaN vertical device. Then, the following epitaxial layers show a drastic polarization effect. However, in the opposite direction of the Ga-polar counterparts, the homoepitaxial layers along the *N*-polar direction demonstrate unique device properties because of the higher decomposition temperature and polarity-dependent property [45, 46]. With the *N*-polar single-crystal FS-GaN substrate, an electric field of 2.2 MV/cm without any edge terminations was achieved, as well as a *BV* of 2.4 kV with N_2_O surface plasma treatment and field plate (FP) [46, 47]. As another special GaN substrate, the *m*-plane also attracts wide attention given its nonpolar property for which the Ga:N was 1:1 in the *m*-plane with respect to only Ga in the *c*-polar and only *N* in the *N*-polar substrates. With this nonpolar GaN substrate, the characteristics of PNDs were reported with the critical electrical field of 2.0 MV/cm and high on/off ratio with no FP or edge termination [48].

### N-GaN Epitaxial Techniques

Prior to the existence of high-quality conductive GaN substrates, GaN PNDs were mainly fabricated on foreign substrates including Si, SiC, and sapphire. Therefore, the structure of devices was always limited to lateral ones. A method based on the lateral structure was proposed to improve the epitaxial quality. Given low threading dislocation (TD) on the window region at lateral devices, the GaN epitaxial layer grown laterally across the mask can achieve a much lower TD density. Thus, lateral epitaxial overgrowth was performed to grow the GaN epitaxial layer on a sapphire substrate for lateral PNDs. The reverse leakage current was suppressed by three orders of magnitude [49].

With the development of epitaxial technology, high-quality single-crystal GaN substrates with low dislocation densities of 3 × 10^–6^ cm^−2^ became available, and GaN PNDs were fabricated on GaN substrates. Combined with upper epitaxial layers by MOCVD grown, the devices show a low leakage current and high *BV* of 600 V to 4 kV corresponding to drift thickness from 6 to 40 μm [16, 50]. With respect to the device on the sapphire substrate, reverse leakage current and *BV* significantly improved [51].

With the native GaN substrate, no special buffer layer usually occurs in the PND epitaxial structure like that on a foreign substrate. The effect of the buffer layer in the PND on the GaN substrate was investigated as reference. The existence of a buffer layer means that the drift layer has lower defect density. Meanwhile, a higher *BV* can be obtained with a thinner drift layer but a thicker buffer layer. The reverse leakage current of the device was improved as well. These device parameters exhibit a strong relationship to the thickness of the buffer layer, which has a slight effect on the forward characteristics of the device [52].

**Fig. 3:**
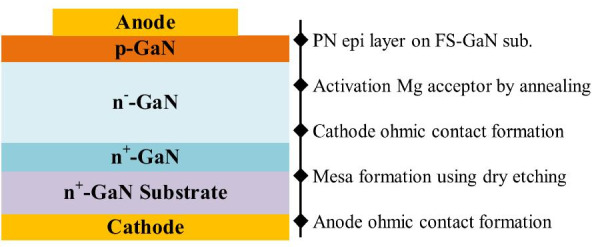
Schematic structure and fabrication process of GaN vertical PND

The upper epitaxial layers are deposited by the MOCVD, metal organic vapor phase epitaxy (MOVPE), and MBE, as discussed in this portion. The epitaxial structure of PNDs includes a highly doped *n*^+^-GaN layer and a drift layer of *n*^−^-GaN between the substrate and *p*-GaN (Fig. 3). The highly doped *n*^+^-GaN layer acts as a transition layer on the conductive GaN substrate in some reports.

In general, the *BV* determines the application scenarios of devices. For example, the higher *BV* for a SiC device relative to that with GaN allows the SiC device to be utilized in high-power and high-voltage applications like electric vehicles and bullet trains (Fig. 1). Similarly, PN diodes are usually applied in higher voltages with respect to the Schottky diode and HEMTs. In PNDs, the *BV* is mainly undertaken by the drift layer (lightly doped *n*^−^-GaN layer) with the broadening of the depletion region (as represented in Eq. 1 with applied voltage *V* for which *V* is positive/negative for the forward/reverse bias when the device is under reverse bias. Moreover, the breakdown occurs with further increase in the reverse bias.

1 $${W_{\text{D}}} = \sqrt {\frac{{2{\varepsilon_{\text{s}}}\left( {{V_{{\text{bi}}}} - V} \right)}}{q}\frac{{{N_{\text{A}}} + {N_{\text{D}}}}}{{{N_{\text{A}}}{N_{\text{D}}}}}}$$

where *S*, *q*, *V*_bi_, *N*_D_, and *N*_A_ are the area of the junction, electronic charge, built-in potential, donor concentration, and acceptor concentration, respectively [53].

According to the depletion width, the devices can be divided into nonpunch-through [*W*_D_ > *W*_Dm_ (*W*_Dm_: maximum depletion-layer width)] and punch-through (*W*_D_ < *W*_Dm_). Given the issues of epitaxy structure and edge, most GaN vertical devices fall under the punch-through type, i.e., their depletion regions expand beyond the entire *n*^*−*^-GaN layer and reach the *n*^+^-GaN substrate prior to breakdown.

Moreover, as described in Eq. (2), the *BV* can be enhanced by increasing the thickness and decreasing the *n*-type doping concentration of the drift layer.

2 $${\text{BV}} = {E_{\text{C}}}t - \frac{{q{N_{\text{D}}}{t^2}}}{{2{\varepsilon_0}{\varepsilon_{\text{r}}}}}$$

where *E*_C_, *q*, *t*, *N*_D_, and *ε*_r_ are the critical electric field, electron charge, *n*^*−*^-GaN layer thickness, net carrier concentration of the *n*^*−*^-GaN layer, and semiconductor permittivity, respectively [54]. Fortunately, experience indicates that a drift layer with thicker thickness and lower doping contributes only to a slight increase for on-state resistance in forward conduction.

Compared with enhancing the *BV* by increasing the drift layer thickness, decreasing of the *n*-type doping concentration in the *n*^*−*^-GaN layer is currently difficult, because of the high background concentrations including those of Si, O and so on. For further decrease in the doping concentration in the *n*^*−*^-GaN layer, the unintentional doping concentration is investigated. Many research groups introduced a nominal i-GaN approximate 10^15^–10^16^ cm^*−*3^ contacting *p*-GaN to form the *p*–*i*–*n* structure to improve the BV of diodes [55].

Certainly, the precondition of these measures is a high epitaxial quality of the drift layer owing because the TDs can increase the off-state leakage and degrade the *BV* properties [56].

To further improve the epitaxial quality of the drift layer in GaN vertical PNDs, growth by MBE was investigated on the GaN substrate. A leakage current of less than 3 nA/cm^2^ and electric field of 3.1 MV/cm were obtained by the very low dislocation density. The nearly ideal breakdown performances indicate that the MBE is an effective method for growing epitaxial layers in GaN vertical PNDs [56]. However, similar to ammonothermal growth, the epitaxial rate is another disadvantage.

### P-GaN Epitaxy

P-GaN, an important component in GaN vertical PNDs, was reported in 1989 since the appearance of GaN by HVPE in 1969 [57, 58]. It was first utilized to obtain blue LED. Then, the *p*-GaN was gradually employed at electric devices field such as normally-off devices and PNDs. In vertical GaN PNDs, the *p*-GaN constitutes light (around 10^19^ cm^−3^) and heavy (≥ 10^20^ cm^−3^) doping concentration, which correspond to forming a PN junction layer with the drift layer and facilitating ohmic contacts as the anode electrode.

Generally, the epitaxial growth of a *p*-GaN is fabricated by MOCVD at a temperature of about 1000 °C and adopts the Mg^2+^ as acceptor. Then, the grown *p*-GaN layer must be activated at high temperatures of 700–800 °C in N_2_ ambient or 400 °C in O_2_ ambient, thereby facilitating a relatively high hole concentration [59–61]. However, the high ionization energy of 150–200 meV of Mg-H bond means a *p*-GaN activation rate of only 1–3%. A low activation rate is also related to the temperature. The Mg dopants can be re-passivated in high temperatures of ≥ 600 °C in NH_3_ or hydrogen ambient [62]. To improve hole concentration, solely raising the doping concentration is not feasible because a higher doping concentration in the *p*-GaN could lead to a deterioration of the crystalline quality of the layer and reduce the hole density through a self-compensation effect [63]. At present, a peak of hole density can usually be achieved at an acceptor concentration of approximately 3 × 10^19^ cm^−3^ [64]. Although being some issues for *p*-GaN, related investigation reports are not much. It may be ascribed to two reasons. One is the limitations of the intrinsic properties of material and epitaxial equipment. Another is that the present *p*-GaN can also obtain comparative device performance.

However, the optimization of the growth condition and novel epitaxial process are still being investigated. For example, in 2017, the non-activated regrown *p*-GaN by MBE was proposed and has an advantage over MOCVD. With the low hydrogen growth environment, a *BV* of 1.1 kV and ideality factor of 2.5 were achieved by the fabricated vertical GaN PNDs [65]. Another interesting *p*-GaN fabrication method is Mg ion implantation with an ultra-high-pressure annealing (UHPA) process. The results reveal that a high activation ratio of 70% and hole mobility of 24.1 cm^2^V^−1^ s^−1^ were achieved with a post-implantation annealing at temperature of 1573–1753 K in N_2_ pressure of 1 GPa. This result is comparable with that of epitaxy growth by MOCVD [66].

In conclusion, for substrate, the commercial low defect GaN substrate is now available. Meanwhile, epitaxial technology is still developing, a situation which strongly affects on device performances. In comparison with MOCVD, the MBE can achieve excellent epitaxial quality, thereby resulting in great device performance without any edge terminations. However, its slow growth rate and high cost render the MBE unfit for large-scale productivity. The epitaxy growth by MOCVD is still the main productive method in practice. Thus, improving the quality of epitaxial layers grown by MOCVD is an urgent issue, which would take considerable time. Therefore, advanced device structure design or measures are proposed for great performance at this stage.

## Edge Termination Techniques

For a vertical GaN PNDs, *BV* is an important parameter. Almost all investigations on vertical GaN PNDs are centered on improving the withstanding voltage at reverse (i.e., *BV*). However, due to the electric field crowding at the edge of PN junction, the depletion layer edge, or the electrode edge, premature breakdown often occurs. Therefore, to reduce the electric field crowding of the device, advanced device structure designs (i.e., edge termination techniques) are developed. Varied edge termination techniques have now been adopted to relax the electric field crowding at the edge of GaN PNDs for a higher *BV*. These techniques include mainly the field plate (FP), ion implantation and plasma treatment, and mesa etching in varied angle or steps and guard rings (GRs) [49, 67–69]. These features are discussed in this section.

### Metal Field Plates

The FP has been widely utilized in GaN-based devices for transferring the peak electric field far from the edge of the gate, anode, or junction. This method has an identical feature to relax the intensity of the electric field at the PN edge under reverse bias. Moreover, the low leakage current and high breakdown voltage under reverse voltage can be achieved by using FP termination, which has a relatively simple fabricating process as well.

**Fig. 4:**
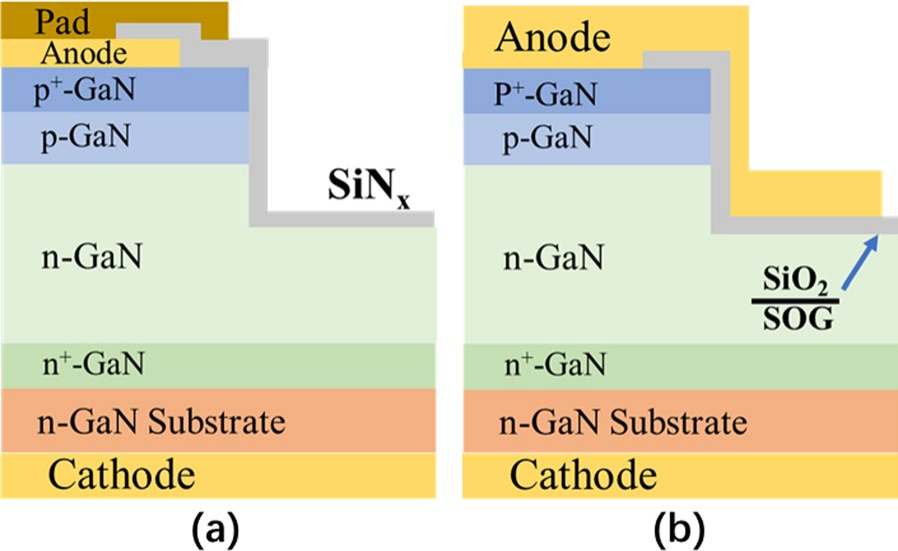
PNDs’ structures with **a** FP and extended passivation dielectric layer and **b** extended FP metal and passivation dielectric layer [70, 71]

The merits of the FP structure include a simple fabrication process and compatibility with the device process. Simultaneously, the dielectric layer of FP is also the passivation layer of the device. As the earliest and most widely used edge termination for vertical GaN PNDs, the non-extended FP termination was initially used, as shown in Fig. 4a. For fabricating the FP structure, mesa structures were processed by inductively coupled plasma (ICP) dry etching. Then, to suppress the parasitic leakage currents from plasma damage, a passivation dielectric film was deposited all over the anode electrode and the entire mesa structure [70].

To furtherly relax the electric field crowding at the periphery of the PN junction edge, the extended metal of the FP was utilized to cover the entire mesa to reduce the reverse leakage current and raise the *BV* [71]. This optimized FP structure is now widely employed in vertical GaN PNDs fabrications. As shown in Fig. 4b, the *BV* was raised over 3 kV with an extended FP structure. Meanwhile, the reverse leakage current was also suppressed to a quite low level at revere voltage of 3 kV.

As an essential component of the FP structure, the passivation layer has a considerable impact on the device characteristics. Thus, an appropriated passivation layer is essential. A passivation layer owned high-*k* permittivity is proposed by simulation, and uniform reverse current flow can be also obtained [50]. According to this theory, vertical GaN PNDs with FP termination using Ga_2_O_3_ (the dielectric permittivity of 10) as passivation film were reported. The *BV* had a large improvement from 200 to 550 V, thereby revealing that a high-k permittivity film such as Ga_2_O_3_ is promising as a passivation film of FP termination in vertical GaN PNDs for raising device characteristics [72]. However, some demerits arise for FP termination. The main issue is the defect during the dielectric layer deposition and interface between the dielectric and GaN, which result in carrier trapping. These would lead to the instability of device performance during long-term use. Therefore, the optimized deposition process of the dielectric layer must be investigated.

### Mesa Termination

**Fig. 5:**
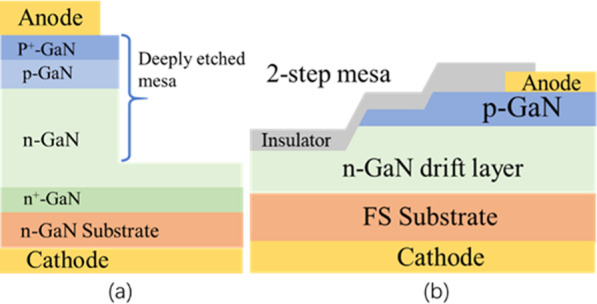
**a** PNDs with deeply etched mesa structure; **b** PNDs with two-step mesa [73, 74]

Mesa etching is an indispensable step to isolate adjacent devices in the fabrication of planar GaN-based devices. Given the simple process, this structure is popular for vertical GaN PND processes. Aside from a uniform electric field at the edge of PN junction, a high *BV* with nondestructive and avalanche characteristics can be achieved in PNDs. For instance, a simple but deep mesa structure can obtain great performance. As shown in Fig. 5a, with more than 10 µm depth of mesa structure in vertical PNDs, nondestructive *BV* and avalanche characteristics were confirmed [73].

The novel mesa structures are investigated as shown in Fig. 5b. A multi-mesa (two-mesa) structure was adopted in vertical PNDs. With respect to the common single mesa structure, the two-mesa structure can shift the peak electric field from the edge of the PN junction to underneath the thinned *p*-GaN in the upper mesa because of the total depletion of holes in the thinned *p*-GaN layer. A high *BV* of 4.7–4.8 kV with nondestructive feature was successfully achieved by the two-mesa structure. Avalanche capability was obtained without lowering the forward *I*–*V* characteristics [74]. On the other hand, the two-mesa structure has the identical function for quasi-vertical PINDs [75]. The *BV* was enhanced from 665 to 835 V with the low leakage current simultaneously.

**Fig. 6:**
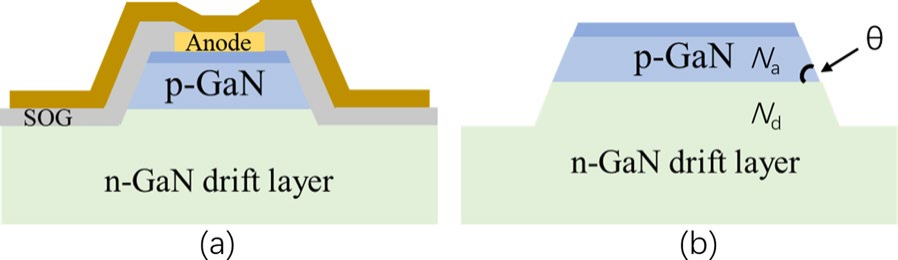
Schematic cross of PND structure with **a** bevel mesa and FP structure, **b** PND epitaxial structure is simulated by treating *N*_A_, *N*_D_ and *θ* as variable [76, 77]

Besides the common perpendicular mesa structure, a mesa structure with negative bevel was recently proposed to mitigate the electric field crowding at edge of the PN junction. With the negative bevel mesa, the electric field at the edge has a decreasing trend when the bevel angle *θ* is lowered from 90˚*.* The peak electric field would be transferred into device inner. In [76], experimental investigation showed that beveled mesa structure (Fig. 6a) could induce a higher *BV* over 3 kV and a low leakage current with respect to *BV* of 3 kV in PNDs with steep mesa when using the same FP structure. Further investigation was performed by simulation using technology computer-aided design (TCAD). In vertical GaN PNDs with beveled mesa (Fig. 6b), the maximum electric field was determined by the acceptor concentration *N*_A_ in *p*-GaN, donor concentration *N*_D_ in *n*^−^-GaN drift layer, and *θ* of the beveled mesa. By theoretical analysis and simulation, the smaller *θ* could lead to higher *E*_pp_ (parallel-plane breakdown field). At the same time, a lightly doped *p*-GaN is beneficial for achieving high *BV* for a fixed *θ* of the beveled mesa. Taking *θ* = 10° as an example, the experimental results support these findings. A parallel-plane breakdown field of 2.86 MV/cm was achieved, and this outcome is consistent with the simulation [77].

Similar with FP termination, the dry etching and/or insulator (or passivation film) is required in process of edge termination. Thus, the damage from dry etching must be considered and can cause a high reverse leakage current, even the premature breakdown. At the same time, it is a high demand to etched equipment for etched precision, and the etched depth is difficult to control. Meanwhile, the existence of damage generates a more complicated interface state between the insulator and the etched semiconductor, a situation that would affect device reliability. Consequently, reducing or alleviating damage is an inevitable issue. Now, some recipes (e.g., tetramethylammonium hydroxide (TMAH) and post-annealing that have the features of removing the damage by wet etching and repairing dry etching damage, respectively) have been adopted to treat etched surface to improve performance.

### Ion Treatment

Ion implantation was employed to alleviate the electric field concentration near the edge. As a relatively simple termination structure, an implantation-based technique was investigated in GaN devices, which includes the compensating species (e.g., O, H, and Zn) or inert species (e.g., Ar, N, He, and Kr) to create deep-level traps in the termination regions [78–82]. Recently, for moderating the occurring of premature breakdown in vertical GaN PNDs, the ion implanted termination (e.g., N, F, Mg, and H) is also utilized. However, the mechanisms of these ion implanted terminations are different.

**Fig. 7:**
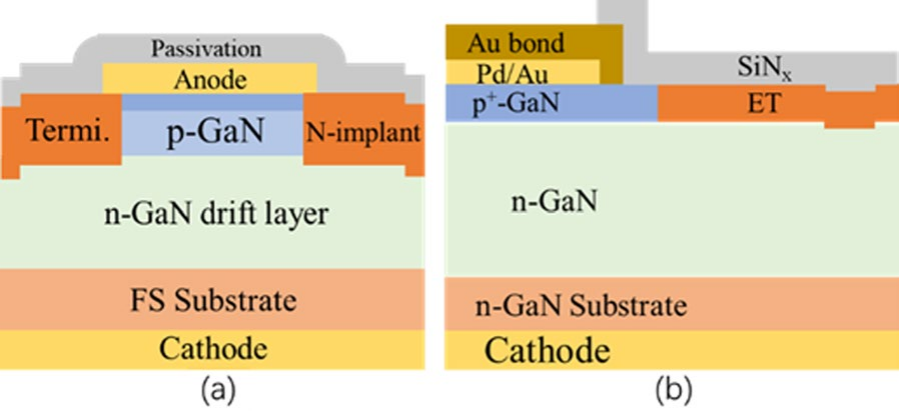
**a** Cross-sectional plot of the PNDs with *N* implantation termination, and **b** *N* implantation termination with fully and partially compensated [53, 84]

With respect to vertical GaN SBDs [83], N implantation in vertical GaN PNDs reveals a different mechanism, which entails creating the donor-like defects (*N* vacancy and *N* interstitial) to compensate for the *p*-type dopants. After processing by *N* implantation as shown in Fig. 7a and b, the conductivity of *p*-GaN can be reduced or eliminated (insulating) by N implantation [53]. To further improve the *BV*, a non-fully compensated layer was proposed, that is, a fully compensated layer coupled with a partially compensated counterpart in *p*-GaN. Thus, a higher *BV* was expected, and the mechanism was analyzed by simulation [84]. The experimental results show that N implantation with partially compensated *p*^+^-GaN could induce a *BV* of 1.68 kV without compromising the forward characteristics [85].

**Fig. 8:**
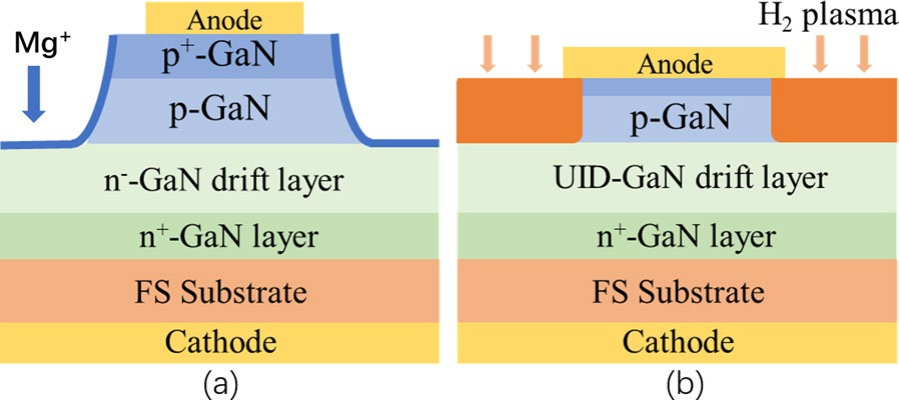
**a** Schematic view of PND structure by Mg ion treatment; **b** PNDs with H_2_ plasma treatment [86, 88]

The hydrogen-plasma (H-plasma) edge termination can also reduce *p*-GaN conductivity, but with another mechanism. H-plasma treatment is an effective passivation method to transform the conductive *p*-GaN into a highly resistive one because of the strong bond of Mg-H in *p*-GaN. In contrast to N implantation, the H-plasma treatment is more appropriate for use in vertical GaN PNDs because of the low damage, low temperature, and simple operation involved. As shown in Fig. 8a, the PNDs exhibited an electric field of 3.0 MV/cm with just the H-plasma treatment. Simultaneously, the devices showed comparable forward *I*–*V* characteristics and a lower reverse leakage current [86].

At the same time, fluorine (F) ion also has the capability of modulating the peak electric field due to the negative fixed charges when the device is under a reverse bias. Nevertheless, if the F implanted edge termination is carried out in vertical GaN PINDs, strict design and structural optimization are needed [87].

In addition to the N, H, and F ion implantation as edge terminations, Mg ion implantation is also an alternative method to convert the surface/interface state, which originates from the damage of ICP dry etching. A novel Mg ion implantation coupled with moat mesa was recently adopted to compensate for the donor-like damage for GaN vertical PNDs (Fig. 8b). Then, a *BV* of 1.5 kV was achieved with a specific on-state *R*_on_ of 0.7 mΩ cm^2^ [88].

Ion implantation termination is an effective method for a high *BV* in vertical GaN PNDs. The ion implantation process is also relatively simple. However, crystal damage occurs during high-energy ion implantation. Moreover, post-annealing at high temperature is required in some of the ion implantation terminations for alleviating the crystal damage. The rectangle-shaped ion implantation profile is pursued, so the implantation depth must be controlled exactly. Finally, although the ion implantation process is simple, the equipment needed is costly due to usage of high energy ion.

### Guard Rings

**Fig. 9:**
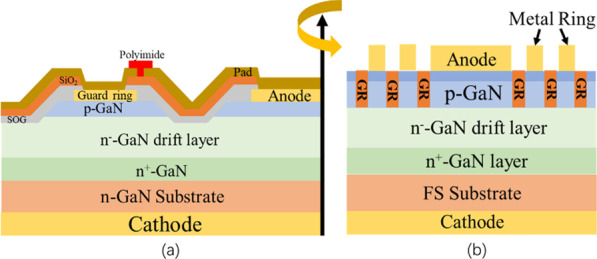
**a** Vertical GaN PNDs structure with floating GR termination; **b** PNDs structure with H-implanted GRs [69, 90]

The use of floating guard rings (GR) as edge termination to improve *BV* is an effective method for vertical GaN PNDs, for which the reverse voltage has a voltage drop over the GR to relax the electric field crowding. At the same time, the GR fabrication process does not require a specialized step, which is synchronous with anode metal deposition. For a higher *BV*, a GR in the FP structure was fabricated in the PNDs (Fig. 9a), in which polyimide was set between the GR and anode portion as resistive device for a further voltage drop. Due to the resistance portion, an incremental 0.2–0.4 kV of *BV* to a maximum of 5.0 kV was obtained. Compared with normal PNDs without a GR structure, the device with a resistive GR exhibited a similar forward *I*–*V* characteristics with *R*_on_ of 1.25 mΩ cm^2^, but a lower reverse leakage current with BFOM of 20 GW/cm^2^ [69].

Besides the floating GR, the H-plasma-based GR structure was also applied to form the edge termination. In addition to low damage, the low diffusivity of H-plasma could result in relatively ideal profile in GaN [89]. Owing to passivation effect of the H-plasma on *p*-GaN, the conductive *p*-GaN becomes highly resistive, and the GR structure was subsequently formed by the highly resistive *p*-GaN ring (Fig. 9b). The narrow GR width of 1–2 µm and decreasing spacing were used to improve the *BV.* GR structures with varied rings were measured. The PNDs with varied rings have similar forward characteristics with around 0.65 mΩ cm^2^ and an ideality factor of 1.65 compared with those without GR. More GRs could further relax the electric field at the device edge. Thus, the devices exhibited a higher *BV* by increasing the number of rings after measurement. Consequently, the PNDs showed a *BV* of 1.7 kV with the highest electric field of 3.43 MV/cm 10 GRs [90].

The disadvantage of GR is its low area utilization rate, and the GR structure generally requires a large area, which is even bigger than anode. Then, the amount of GR raises the design difficulty such as the width and spacing of the GR. At present, two and more kinds of edge termination are adopted in single vertical GaN PNDs for a higher *BV*. These edge terminations typically include the FP, bevel mesa, and guard rings due to the relatively simple fabrications. On the other hand, these edge terminations are not meant to introduce the foreign element into the GaN crystal with respect to ion treatment, a feature that is beneficial for device performance. However, the quality of epitaxial layers still occupies a considerable proportion.

## Vertical GaN PNDs on Si Substrate

To date, although vertical GaN-on-GaN devices exhibit excellent performance, the high cost and small diameter of GaN substrates still impede their large-scale market applications. Consider to the merit of a large scale and low cost of Si substrate, GaN-on-Si devices have attracted considerable many attentions at this stage.

**Fig. 10:**
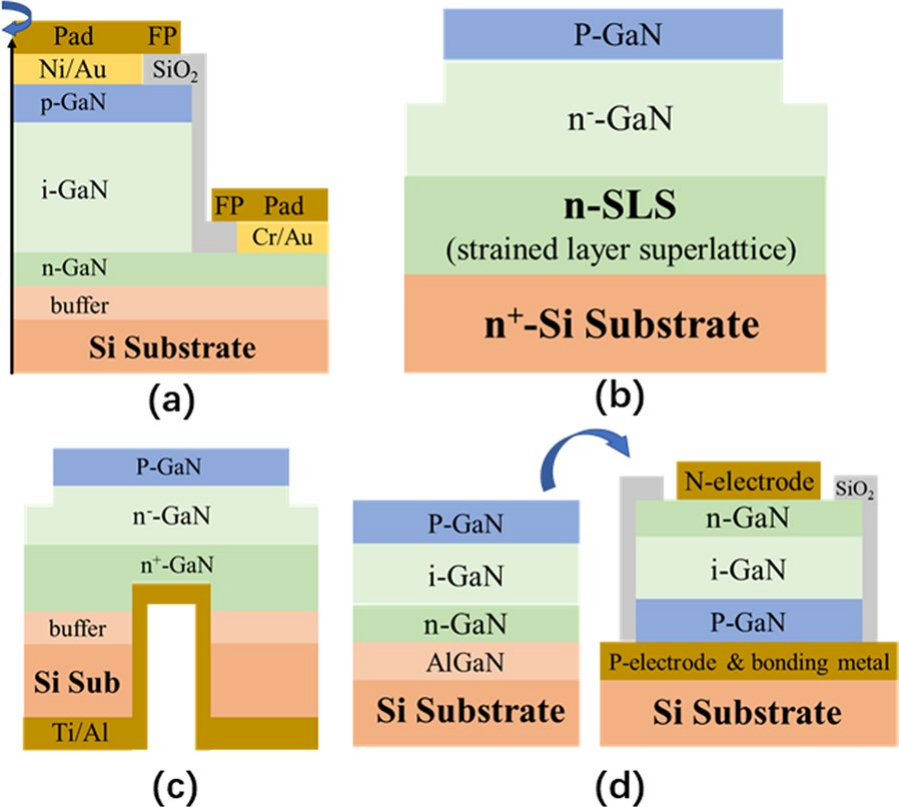
**a** Quasi-vertical PINDs with FP; **b** fully vertical PNDs by using the thin AlN and SLS superlattice structure on conductive Si substrate; **c** PNDs with trenched ohmic contact on back; **d** PNDs fabricated by layer transfer technology [91–94]

For the quasi-vertical PND on Si substrate, high-quality buffer is essential. In [91], as shown in Fig. 10a, optimizing the AlN nucleation layer and the succeeding growth process, a GaN drift layer with a low threading dislocation density of 2.95 × 10^8^ cm^−2^ and high electron mobility of 720 cm^2^/Vs was obtained. With the FP structure, the device has a *BV* of 820 V with *R*_on_ of 0.33 mΩ cm^2^.

In addition to the challenge of materials mismatch, the conductive buffer layer and Si substrate are fundamental for fully vertical PNDs on Si substrate. In Fig. 10b, a *n*^+^-type Si substrate was first utilized as the cathode of vertical PNDs. Subsequently, the Si-doped thin AlN layer and GaN/AlN strained superlattice layer were employed to facilitate device performance [92]. Vertical PNDs on Si substrate are proposed with FP, and a *BV* of 288 V is confirmed with a drift layer of 1.5 µm. Moreover, a higher *BV* of 720 V in PNDs on Si with 5.7 µm drift layer is reported in Fig. 10c [93]. Unlike the conductive Si substrate method, the cathode ohmic on back approach was made by a trench, which reaches the *n*^+^-GaN layer through the selective removal of regular Si substrate and buffer layer.

Besides the conductive Si substrate and trenched ohmic contact on back, substrate removal technology is an alternative method to produce vertical GaN PNDs on Si substrates. In Fig. 10d, a layer transfer technology is used to process vertical PINDs on Si substrate. After Si substrate removal, transfer, *n*-electrode, and sidewall passivation, the devices exhibit a low *R*_on_ of 3.3 mΩ cm^2^ and *BV* of 350 V [94]. The high BFOM value of 37.0 MV/cm^2^ in PINDs demonstrates that substrate removal technology is an effective way for GaN-based PIND fabrication on Si substrates. Relative to other technologies, however, the more complicated fabrication process and higher production cost are issues that must be addressed during the substrate removal part.

As an alternative technical route, PNDs on Si substrates are fabricated with quasi-vertical or vertical device structures. To mitigate the substantial material mismatch between the Si substrate and GaN epitaxial stacks, a more effective epitaxial technology must be investigated. For a higher *BV*, thicker drift layer is also essential for a higher *BV*. This fact presents another key issue to investigate because the drift layer thickness on Si substrate is approximately 5 µm.

## Future Challenges and Conclusion

Vertical PNDs are essential to simultaneously obtain high current (> 100 A) with high voltages (> 600 V), which can meet the requirements of several applications including electric vehicles and recycled energy processing. Despite the great progress achieved, applications of vertical GaN PNDs remain several barriers such as cost and technical limitations.

For FS-GaN substrate, high epitaxial quality with low threading dislocation has been achieved by a common MOCVD. The small size and high cost of the FS-GaN substrate confine the applications of the vertical GaN PNDs within a narrow range, and the small size also raises the price of GaN. The commercial GaN substrate is only 2-inch and is much smaller than 4–6 inch SiC and 8–12 inch Si substrates. The primary challenge for vertical GaN PNDs is achieving high epitaxial quality, especially the high quality of the *p*-GaN layer with a high hole concentration. Recently, novel PNDs are proposed through replacing *p*-GaN with NiO_*x*_ synthesized by thermal oxidation or sputtering; great performance is also demonstrated as replacement of *p*-GaN in vertical PND and guard ring in GaN SBD [95–98].

**Fig. 11:**
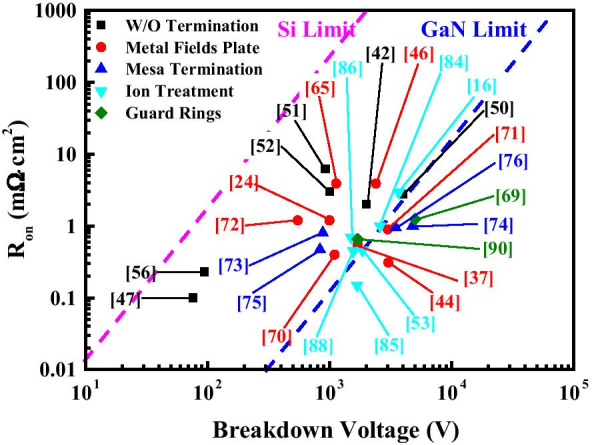
Benchmarking of the *R*_on_ versus *BV* of quasi- or full-vertical GaN PNDs

Vertical GaN PNDs, as one of the most promising power devices, are reviewed and summarized in this study. The recent progresses on vertical GaN PNDs, including comparison of different materials (SiC vs. GaN) and different device structures (SBD versus PND), material epitaxy growth and edge termination techniques (FP, MESA, ion implanted and GR edge terminations), are discussed. The values of *R*_on_ versus *BV* by varied technologies are plotted in Fig. 11. Aside from the epitaxial technologies, edge termination technologies play a key role for vertical GaN PNDs to achieve high device performance at this stage. Despite its great progress in terms of device performance, the advantages of vertical GaN PNDs remain under-exploited. The characteristics of vertical GaN PNDs could be promoted by optimizing the device structure and epitaxial quality in succeeding studies. Then, commercialized vertical GaN PNDs will soon be available in the future with mature edge termination and epitaxial techniques.

## Data Availability

The datasets used or analyzed during the current study are available from the corresponding author on reasonable request.

## Abbreviations

GaN: gallium nitride
SiC: silicon carbon
PND: PN junction diode
PIND: P-i-N junction diode
SBD: Schottky barrier diode
IGBT: insulated gate bipolar transistors
BJT: bipolar junction transistor
MOSFET: metal oxide semiconductor field effect transistor
BFOM: Baliga’s figure of merit
HFET: heterostructure field-effect transistor
JFET: junction field-effect transistor
2DEG: two-dimensional electron gas
BV: breakdown voltage
FS: free-standing
MOCVD: metalorganic chemical vapor deposition
MBE: molecular beam epitaxy
JBS: junction barrier Schottky
MPS: merged PN Schottky
LED: light-emitting diode
HVPE: hydride vapor phase epitaxy
FP: field plate
TD: threading dislocation
MOVPE: metalorganic vapor phase epitaxy
HEMT: high electron mobility transistor
UHPA: ultra-high-pressure annealing
GR: guard ring
ICP: inductively coupled plasma
TCAD: technology computer-aided design
TMAH: tetramethylammonium hydroxide
